# Permeability of *Hypogymnia physodes* Extract Component—Physodic Acid through the Blood–Brain Barrier as an Important Argument for Its Anticancer and Neuroprotective Activity within the Central Nervous System

**DOI:** 10.3390/cancers13071717

**Published:** 2021-04-05

**Authors:** Elżbieta Studzińska-Sroka, Aleksandra Majchrzak-Celińska, Przemysław Zalewski, Dominik Szwajgier, Ewa Baranowska-Wójcik, Marcin Żarowski, Tomasz Plech, Judyta Cielecka-Piontek

**Affiliations:** 1 Department of Pharmacognosy, Poznan University of Medical Sciences, Święcicki 4 Str, 60-781 Poznań, Poland; pzalewski@ump.edu.pl (P.Z.); jpiontek@ump.edu.pl (J.C.-P.); 2 Department of Pharmaceutical Biochemistry, Poznan University of Medical Sciences, Święcicki 4 Str, 60-781 Poznań, Poland; majchrzakcelinska@ump.edu.pl; 3 Department of Biotechnology, Microbiology and Human Nutrition, University of Life Sciences in Lublin, Skromna 8 Str, 20‐704 Lublin, Poland; dominik.szwajgier@up.lublin.pl (D.S.); ewa.baranowska@up.lublin.pl (E.B.-W.); 4 Department of Developmental Neurology, Poznan University of Medical Sciences, Przybyszewski 49 Str, 60-355 Poznań, Poland; zarowski@ump.edu.pl; 5 Department of Pharmacology, Medical University of Lublin, Chodźki 4a Str, Lublin, Poland; tomasz.plech@umlub.pl

**Keywords:** *Hypogymnia physodes*, anticancer, chemopreventive and neuroprotective activity, blood–brain barrier permeability

## Abstract

**Simple Summary:**

Central nervous system (CNS) diseases, including tumors such as glioblastomas and neurodegenerative diseases, such as Alzheimer’s disease, are some of the greatest challenges of modern medicine. Therefore, our study aimed to evaluate the anticancer and neuroprotective activity of the extract from a common European lichen *Hypogymnia physodes* and of its compound-physodic acid. The examined substances were cytotoxic against the glioblastoma cell lines A-172, T98G, and U-138 MG. Both substances strongly inhibited hyaluronidase, and diminished cyclooxygenase-2 activity (*H. physodes* extract), enzymes expressed in patients with malignant glioma. Furthermore, *H. physodes* extract inhibited tyrosinase activity, the enzyme linked to neurodegenerative diseases. The tested substances exhibited antioxidant activity, however, acetylcholinesterase and butyrylcholinesterase inhibitory activity were not high. We proved that physodic acid can cross the blood–brain barrier. We conclude that physodic acid and *H. physodes* extract should be regarded as promising agents with anticancer, chemopreventive, and neuroprotective activities, especially concerning CNS.

**Abstract:**

Lichen secondary metabolites are characterized by huge pharmacological potential. Our research focused on assessing the anticancer and neuroprotective activity of *Hypogymnia physodes* acetone extract (HP extract) and physodic acid, its major component. The antitumor properties were evaluated by cytotoxicity analysis using A-172, T98G, and U-138 MG glioblastoma cell lines and by hyaluronidase and cyclooxygenase-2 (COX-2) inhibition. The neuroprotective potential was examined using COX-2, tyrosinase, acetylcholinesterase (AChE), and butyrylcholinesterase (BChE) activity tests. Moreover, the antioxidant potential of the tested substances was examined, and the chemical composition of the extract was analyzed. For physodic acid, the permeability through the blood–brain barrier using Parallel Artificial Membrane Permeability Assay for the Blood–Brain Barrier assay (PAMPA-BBB) was assessed. Our study shows that the tested substances strongly inhibited glioblastoma cell proliferation and hyaluronidase activity. Besides, HP extract diminished COX-2 and tyrosinase activity. However, the AChE and BChE inhibitory activity of HP extract and physodic acid were mild. The examined substances exhibited strong antioxidant activity. Importantly, we proved that physodic acid crosses the blood–brain barrier. We conclude that physodic acid and *H. physodes* should be regarded as promising agents with anticancer, chemopreventive, and neuroprotective activities, especially regarding the central nervous system diseases.

## 1. Introduction 

Bioactive compounds from natural sources can be regarded as important agents that can potentially be applied in the prevention and treatment of human diseases. Many phytochemicals, e.g., resveratrol, curcumin or epigallocatechin gallate, inhibit pathological processes, thus protecting against the development of various human diseases [1,2,3]. Over the last 10 years, a dynamic increase in the rate of the central nervous system (CNS) diseases has been observed. Delicate alterations in the precise mechanisms regulating CNS homeostasis may lead to the progressive loss of structure or function of neurons, which is present in neurodegenerative diseases such as Alzheimer’s (AD), or can cause malignant transformation of cells, leading to brain tumors. Glial tumors are especially formidable, since they are not only the most common primary brain tumors, but also the most aggressive ones—the median survival of glioblastoma, the most common type of glial tumor, is around 15 months [4] and has not measurably decreased despite huge scientific efforts and novel treatment modalities. Neurodegenerative diseases, such as AD or Parkinson’s disease (PD), are also significant public health care challenges. They are understood as a chain of events leading to a gradual loss of neurons’ functional properties and leading to the death of the nerve cells [5]. Due to the aging profile of society, they are currently one of the most critical therapeutic problems. There are no effective therapies for the illnesses mentioned above. Therefore, substances influencing their pathological mechanism, which could facilitate effective treatments, are constantly being sought and tested.

Various mechanisms are important in the development of CNS pathological changes. Oxidative stress and many other processes (for example, inflammation, mitochondrial dysfunction, apoptosis) are considered the cause of CNS diseases [4,6]. Reactive oxygen species (ROS), resulting among others, from the metabolic transformations of neurotransmitters can attack sensitive glial cells and neurons, leading to the damage of CNS structures [7,8].

Hyaluronic acid (HA), anionic, nonsulfated glycosaminoglycan, is the main component of the brain extracellular matrix. It is synthesized by the hyaluronan synthase (HAS) family and degraded by hyaluronidase [9]. The degradation of HA by hyaluronidase can produce final fragments with different molecular weights. In glioblastoma, fragments <20 kDa and between 20 and 50 kDa are the most abundant isoforms [9]. These isoforms were found to be associated with enhanced cancer cell proliferation and invasion capacities, as well as proinflammatory and proangiogenic properties [10].

Moreover, most brain tumors, including glial tumors of different grades (e.g., glioblastoma, astrocytoma, oligodendroglioma), and medulloblastoma, craniopharyngioma, ependymoma and neurinoma, express high levels of cyclooxygenase 2 (COX-2). In glioblastoma, COX-2 expression levels were found to correlate with many aggressive aspects of the disease, such as proliferation rate, glioma WHO grade, and poor prognosis [11]. Thus, COX-2 can also be regarded as a crucial target in brain tumor therapy.

Tyrosinase is another enzyme which is linked to neurodegenerative diseases, such as PD. It oxidizes the excess dopamine to produce dopamine quinones, highly reactive species which induce neural damage and cell death [12]. Therefore, tyrosinase inhibition is anticipated to provide a new therapy for neurodegenerative disorders.

Other enzymes important for proper CNS functions are acetylcholinesterase (AChE) and butyrylcholinesterase (BChE), both hydrolyzing a neurotransmitter-acetylcholine. Inhibition of acetylcholine hydrolysis is beneficial in neurodegenerative disorders, such as AD. Moreover, noncholinergic functions of AChE and BChE, such as regulation of proliferation processes and impact on cellular adhesion are also essential in brain tumors.

An important criterion for achieving action within the CNS is the penetration of compounds through the blood–brain barrier. Among the mechanisms of xenobiotics penetration through biological barriers, the prominent role is played by passive transport, which, according to the literature data, concerns 80–95% of substances [13]. Many natural substances are absorbed through passive transport, including flavonoid aglycones (e.g., naringenin) [14], anthocyanidins [15], as well as compounds present in fungi and lichens, usnic acid (dibenzofuran) [16], or norlobaridone (depsidone) [17].

Lichens are an important source of bioactive compounds with interesting and little-known biological activities. The antimicrobial activity of lichens, their antioxidant, enzyme-inhibiting, and anti-inflammatory properties were studied [18,19]. Their antitumor activity was also proved and tested in cancer cell lines and animal cancer models [20]. The neuroprotective properties of lichen compounds are a relatively new issue. The studies conducted so far have shown that secondary metabolites of lichens (including depsidones) have the neuroprotective potential. The literature describes fumaroprotocetraric acid (depsidone from *Cetraria islandica*), which, in the human neuroblastoma cell model (SH-SY5Y; neurons) and the astrocytoma cell line model (U373-MG; astrocytes), reduced the cytotoxicity induced by the oxidative stress [21]. The neuroprotective potential of lichens was also associated with the ability to inhibit cholinesterases present in the CNS, thus increasing the neurotransmitter’s level [19].

**Figure 1:**
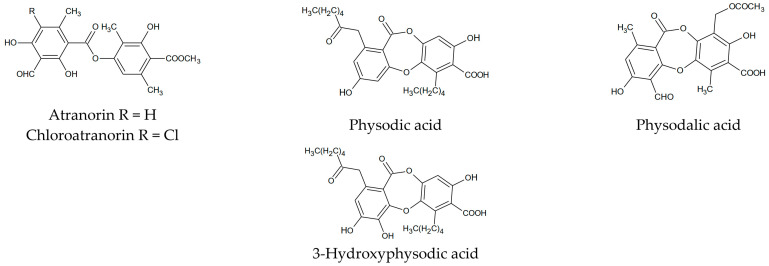
The main compounds present in *Hypogymnia physodes*.

*Hypogymnia physodes,* commonly occurring in Europe, is a lichen from the Parmeliaceae family. This species contains compounds from the group of depsides and depsidones (including physodic acid) (Figure 1), which probably determine its biological potential [22,23,24]. *H. physodes* possess antiproliferative, antioxidant, antibacterial, and anticancer properties [20,25]. Therein, physodic acid was found to inhibit the canonical Wnt pathway in colorectal cancer cells [26] and inhibited M-Phase Phosphoprotein 1 (MPP1), which is upregulated in various bladder cancer types [17].

Regarding the interesting but scant results on antiglioma and neuroprotective activity of lichens, we aimed to screen the biological activity of *H. physodes* acetone extract and physodic acid as the main compound isolated from its extract. The antitumor activity was assessed by examining the cytotoxicity of *H. physodes* acetone extract and physodic acid on A-172, T98G, and U-138 MG glioblastoma cell lines and by examination of hyaluronidase and cyclooxygenase-2 (COX-2) inhibitory properties. The neuroprotective potential was examined by measuring the antioxidant activity and by screening COX-2, tyrosinase, AChE, and BChE inhibition. Moreover, we performed an in vitro PAMPA-BBB assay to assess the ability of physodic acid to cross the blood–brain barrier.

## 2. Materials and Methods

### 2.1. Plant Material

*H. physodes* was manually collected in June 2015 in Jastrzębsko Stare, Poland, and authenticated by Dr. Daria Zarabska-Bożejewicz (The Institute for Agricultural and Forest Environment of Polish Academy of Sciences in Poznan, Bukowska 19, 60-809 Poznań, Poland). A voucher specimen (ES 2015.007) has been deposited in the herbarium of the Department of Pharmacognosy at Poznan University of Medical Sciences, Święcicki 4, 60-781 Poznań, Poland.

### 2.2. Chemicals and Solvents

Formic acid, hydrochloric acid, sodium carbonate, sodium hydroxide, DMSO, acetone, ammonium acetate, copper (II) chloride were purchased from Avantor Performance Materials Poland S.A. (Gliwice, Poland). The Folin-Ciocalteu′s phenol reagent was from Merck (Darmstadt, Germany), HPLC grade water, HPLC grade acetonitrile, and acetate buffer were from the JT Baker–Avantor Performance Materials B.V. (Deventer, The Netherlands), tannic acid from Roth GmbH (Karlsruhe, Germany).

Physodic acid, the mixture of atranorin and chloroatranorin were isolated and identified in the Department of Pharmacognosy of Poznan University of Medical Sciences [26]. Atranorin was purchased from ChromaDex (Los Angeles, CA, USA). All other chemicals were obtained from Sigma–Aldrich (Taufkirchen, Germany).

### 2.3. Preparation of Extract

Dried, cleaned, and fragmented thalli of *H. physodes* (10.0 g) were sonicated at 35 °C for 6 × 30 min with acetone (200 mL × 6) in an ultrasonic bath. The extracts were filtered using Whatman filter paper No. 1 and concentrated by evaporation using a rotary evaporator under vacuum at 35–40 °C to afford a solid residue (1.179 g). The yield of the acetone extract was 11.79%.

### 2.4. Determination of Cytotoxicity of Physodic Acid and H. Physodes Acetone Extract

T98G glioblastoma multiforme cell line was purchased from the European Collection of Authenticated Cell Cultures (ECACC, Salisbury, UK), whereas A-172 and U-138 MG were obtained from American Type Culture Collection (ATCC). These three particular glioblastoma cell lines were chosen for the analysis since they differ in the expression level of the O-6-methylguanine-DNA methyltransferase (MGMT), which is considered as one of the most important predictive biomarkers in glioblastoma patients [27]. These cell lines also differ in the sensitivity to temozolomide, the most commonly used alkylating agent. MGMT is not expressed in A-172, which is therefore regarded as temozolomide sensitive. Contrarily, T98G and U-138 MG cells express MGMT and are temozolomide resistant [28]. All cell lines were cultivated in recommended media, namely, line A-172 was grown in ATCC- formulated Dulbecco’s modified Eagle’s medium (DMEM) (Sigma-Aldrich, St. Louis, MO, USA), whereas T98G and U-138 MG were cultivated in ATCC­formulated Eagle’s Minimum Essential Medium (EMEM) (Sigma-Aldrich, St. Louis, MO, USA), respectively. The media were supplemented with 10% fetal bovine serum (FBS) and 1% antibiotics solution (penicillin and streptomycin). The medium for T98G cell line was additionally supplemented with 2 mM glutamine, 1% nonessential amino acids and 1% sodium pyruvate. For all the experiments, the media containing all the above-listed reagents were used, but the amount of FBS was reduced to 5%. All of the above-mentioned reagents, except FBS, were purchased from Sigma-Aldrich (USA). FBS was obtained from Biowest (Nuaillé, France). Cells from all three cell lines were incubated at 37 °C in an atmosphere consisting of 95% air and 5% CO_2_ in a humidified incubator until they reached 70% confluency, and then they were split.

Physodic acid was dissolved in dimethylsulfoxide (DMSO, Sigma-Aldrich, St. Louis, MO, USA) to make a 20 mM stock solution stored at −20 °C. The dry *H. physodes* acetone extract was diluted in DMSO to make a 10 mg/mL stock solution. For the experiments, the stock solutions were diluted ex tempore to the final selected concentration with the complete cell culture medium containing 5% FBS. The effect of physodic acid on the viability of A-172, T98G and U-138 MG cells was assessed by the MTT assay according to a standard protocol. Cells (1 × 10^4^ per well) were seeded in wells of a 96-well plate, and after 24 h of preincubation, increased concentrations of physodic acid (0.47–47 μg/mL equal 1–100 μM) or *H. physodes* extract (1–100 μg/mL) were added, and cells were grown for 48 h at 37 °C. Cells treated with medium containing a respective concentration of DMSO (Sigma-Aldrich, St. Louis, MO, USA) were used as a control. After 48 h, the cells were washed with 200 μL of PBS, followed by incubation with 3-(4,5-dimethylthiazolyl-2)-2,5 diphenyltetrazolium bromide (MTT) solution (Merck, Darmstadt, Germany) in 10% FBS medium (0.5 mg/mL) for 3 h. After the incubation, MTT solution was removed from the wells, and acidic isopropanol was added. The plates were transferred to the orbital shaker to enhance dissolution of formazan crystals. Finally, the absorbance was measured at λ = 570 nm and λ = 690 nm on the microplate reader (Tecan Infinite M200). All the experiments were repeated three times with four measurements per assay.

### 2.5. Anti-Hyaluronidase Activity

Inhibition of hyaluronidase by the *H. physodes* extract was determined by a method described by Grabowska et al. [29] with minor modifications. Briefly, 25 µL of incubation buffer (50 mM, pH 7.0, with 77 mM NaCl and 1 mg/mL of albumin), 25 µL of enzyme (30 U/mL of acetate buffer pH 7.0), 10 µL solutions of the examined acetone extract (1.875–0.3125 mg/mL) or physodic acid (1.25–0.3125 mg/mL), and 15 µL of acetate buffer (pH 4.5) were combined (the final concentrations were 0.1875–0.03125 and 0.125–0.03125 mg/mL, for extract and physodic acid, respectively). The samples were incubated at 37 °C for 15 min. Next, 25 µL of HA (0.3 mg/mL in acetate buffer) was added. After incubation at 37 °C for 45 min 200 µL of 2.5% cetrimonium bromide (CTAB) in 2% NaOH was added to undigested HA precipitated. The turbidity of the reaction mixture was measured as the absorbance at 600 nm (Multiskan GO 1510, Thermo Fisher Scientific, Vantaa, Finland) after 10 min of incubation at room temperature. Tannic acid was used as the positive control (6.0–1.25 mg/mL, with the final concentration 0.6–0.125 mg/mL). For the investigated substances, three independent experiments were carried out, and the average from n = 5 measurements was calculated. The percentage of inhibition was calculated by using the equation below.

(1) $$\%\;\mathrm{inhibition}\;\mathrm{activity}=\frac{\left(T_{s}-{\mathrm{TE}}_{\mathrm{blank}}\right)}{\left({\mathrm{TH}}_{\mathrm{blank}}-{\mathrm{TE}}_{\mathrm{blank}}\right)}\times 100\%$$

where T_S_—absorbance of sample; TE_blank_—absorbance of the enzyme + examined substance; TH_blank_—absorbance of the HA + examined substance.

### 2.6. Anticyclooxygenase Activity

*H. physodes* extract or isolated physodic acid (prepared as described above) were dissolved in DMSO (for molecular biology, Sigma-Aldrich D8418) to obtain 9.12 mg/mL (456 µg/mL final concentration) and 6.00 mg/mL (300 µg/mL), respectively. These stock solutions were tested undiluted as well as diluted (with DMSO) 1.5-fold and 3-fold. For the assay, reagents from Cayman COX Activity Assay Kit (Chemical, Ann Arbor, MI, USA, No. 760151) were prepared as suggested by the producer and combined with COX-2 enzyme (Human recombinant, Cayman No. 60122, pre-diluted 100-fold using 100 mM, pH 8.0 Tris buffer). A volume of 0.01 mL of a diluted sample was mixed with 0.01 mL hemin, shaken and left for 5 min at 25 °C followed by addition of 0.02 mL colorimetric substrate, 0.02 mL arachidonic acid solution and completed to 0.18 mL using Tris buffer (100 mM, pH 8.0). To start the reaction, 0.02 mL of COX-2 solution was added. The increase of the absorbance during the incubation at room temperature was recorded at 590 nm after 20 min (Tecan microplate reader, Grödig, Austria). Negative (blank) sample (0.01 mL Tris buffer instead of the studied sample) and positive sample (COX-2 inhibitor DuP-697) were run simultaneously. Background of the studied samples (0.01 mL of the sample mixed with 0.19 mL Tris buffer) was also measured and included in the calculations. Each sample was run in at least four repeats.

### 2.7. Anti-Tyrosinase Activity

Tyrosinase inhibitory activity was determined by a spectrophotometric method, as described by Lim et al. [30] with some modifications. A 12.8 mg of the acetone extract from *H. physodes* or physodic acid was weighed, dissolved in 1 mL of DMSO, and used to obtain 6.4 and 3.2 mg/mL concentrations. Next, 25 µL of prepared concentrations was added to 75 µL of 0.1 M phosphate buffer (pH 6.8) and 50 µL of tyrosinase solution (192 U/mL in distillate water). After the incubation at room temperature (25 °C, 10 min), 50 µL of L-DOPA (2 mM) was added and incubated for the next at the same temperature condition (25 °C, 20 min). Each sample was accompanied by a blank that contains all components except L-DOPA. The control sample contained DMSO instead of the test substance. Absorbance was measured at 475 nm. The azelaic acid solution (12.8–3.2 mg/mL prepared in DMSO) was used as the positive control. For the investigated substances, two independent experiments were carried out and the average from n = 4 measurements was calculated. The percentage of tyrosinase inhibition was calculated as:

(2) $$\%\;\mathrm{tyrosinase}\;\mathrm{inhibition}=\frac{A_{\mathrm{control}}-A_{\mathrm{sample}}}{A_{\mathrm{control}}}\times 100\%$$

where A_control_ is absorbance of control sample without blank; A_sample_ is absorbance of tested sample without blank.

### 2.8. Anticholinesterase Activity

Ellman’s colorimetric method was used [31] with some modifications described previously [32]. Tested sample (5 μL, at the concentration of 5 mg/mL—final concentration in the mixture: 72.5 µg/mL) was mixed with 20 μL of AChE (or BChE) solution (0.28 U/mL) and completed after 5 min with 35 μL of ATChI (or BTCh) (1.5 mmol/L), 175 μL of 0.3 mmol/L DTNB (containing 10 mmol/L NaCl and 2 mmol/L MgCl_2_) and 110 μL with Tris-HCl buffer (50 mmol/L, pH 8.0). Samples containing 5 μL of Tris-HCl buffer instead of the studied sample were run in the same way (“blank” samples). The increase in absorbance due to the spontaneous hydrolysis of the substrate was monitored using “blank” samples containing ATCh (or BTCh) and DTNB completed to 345 μL with Tris-HCl buffer. The effect of DMSO on the enzyme activity was tested using 5 μL of DMSO instead of the tested sample. All samples were incubated at 22 °C (30 min, incubation time was determined after optimization experiments, details not shown), and the absorbance was measured (405 nm, 96-well microplate reader, Tecan Sunrise, Grödig, Austria).

The “false-positive” effect of studied compounds was measured according to Rhee et al. [33] with minor modifications, as described previously [32]. After mixing the substrate with the enzyme and buffer, the “false-positive” sample was left for incubation. Then, a studied sample and DTNB were added, followed by an immediate measurement of the absorbance.

Reference cholinesterase inhibitors were used for the calculations of results (eserine, and donepezil). For this purpose, for each compound, 16 dilutions in pure DMSO were prepared (0.09–1.44 μg/mL). These solutions were tested as described above, and calibration curves were calculated.

Each sample was analyzed in at least eight repeats, and all solutions used in a set of analyses were prepared in the same buffer. For calculations, the background of the sample (5 μL mixed with 340 μL of Tris buffer) was measured at 405 nm and subtracted during calculations. Then, the absorbance of the test sample was subtracted from the absorbance of the “blank” sample.

### 2.9. Antioxidant Activity

The CUPric Reducing Antioxidant Capacity (CUPRAC) assay was performed according to Kikowska et al. [34] with modifications. The stock solutions of CUPRAC reagent included equal parts of acetate buffer (pH 7.0), 7.5 mM neocuproine solution in 96% ethanol, and 10 mM CuCl_2_xH_2_O solution. Briefly, 0.05 mL of the dry extract or compound solution dissolved in DMSO at different concentrations (24–96 and 60–1000 μg/mL for the extract and physodic acid, respectively), was mixed with 0.15 mL of CUPRAC solution (the final assay concentrations were 6–24 and 15.0–250 μg/mL, for the extract and physodic acid, respectively), shaken and incubated at room temperature for 30 min in the dark conditions. Then the absorbance was read at 450 nm. Vitamin C and resveratrol were used as a standard (4–64 μg/mL); the final assay concentrations were 1–16 μg/mL, for vitamin C, and 25–400 μg/mL; the final assay concentrations were 6.25–100 μg/mL for resveratrol. For the investigated substances, three independent experiments were carried out, and the average from *n* = 6 measurements was calculated. The results were expressed as the IC_0.5_ which corresponds to the extract concentration required to produce 0.5 absorbance value.

### 2.10. Total Phenolic Content (TPC)

TPC was determined using the Folin–Ciocalteu method [35]. In total, 0.1 mL of the extract, dissolved in DMSO at concentration 2 mg/mL, was mixed with 4.0 mL of distilled water and with 0.5 mL of Folin–Ciocalteu reagent. After 1 min, 2.0 mL of 20% sodium carbonate was added and supplemented with distilled water to a total volume of 10 mL. The absorbance was measured at 760 nm, after 30 min incubation at dark and at room temperature. A blank sample of water and reagents was used as a reference. TPC was expressed as mg of gallic acid equivalent per g of a dry extract (a calibration curve of gallic acid: y = 9.8399x + 0.0289; R^2^ = 0.9993) in a concentration range 0.02–0.08 mg/mL.

### 2.11. High-Performance Liquid Chromatography (HPLC) Analysis

A gradient HPLC method with UV detection was developed and validated for the determination of compounds from extracts. The extracts were dissolved in acetonitrile at concentration 0.4 mg/mL. A Kinetex C18 column (100 × 2.1 mm, 5 μm) was used as the stationary phase. The mobile phase consisted of acetonitrile and 0.5% formic acid with a flow rate of 0.3 mL/min. The initial concentration of acetonitrile was 5%, then it increased to 100% during 10 min. In the next step, isocratic elution with 100% acetonitrile was proceeded for 2 min. Finally, during 5 min the concentration of acetonitrile was decreasing to initial conditions (5%). The detection wavelength was 254 nm, and the temperature was 40 °C. The method was validated with regard to selectivity, linearity, precision, limit of detection, limit of quantitation for physodic acid, and atranorin.

### 2.12. Permeability through the Blood–Brain-Barrier (PAMPA-BBB)

To evaluate the effective permeability (*Pe*) of the *H. physodes* extract and physodic acid, the Parallel Artificial Membrane Permeability Assay (PAMPA) for the Blood–Brain Barrier (BBB) was used (Pion Inc., Billerica, MA, USA). The stock solutions of acetone extract from *H. physodes* and from physodic acid were prepared with DMSO (3 mg/0.1 mL or 1.5 mg/0.1 mL, respectively) and diluted with Prisma buffer (pH = 7.4; from Prisma HT, Pion Inc.) to obtain the donor solutions for physodic acid: 0.45 mg/mL (30 µL of stock/1000 µL of buffer for physodic acid) or 0.9 mg/mL (60 µL of stock/1000 µL of buffer for physodic acid), and 0.9 mg/mL (30 µL of stock/1000 µL *H. physodes* acetone extract). Then, 180 µL of the donor solution were added to the donor wells. Subsequently, each filter membrane of the top plate was coated with 5 μL BBB-1 lipid solution (Pion Inc.) and the acceptor well was filled with 200 µL BSB (Brain Skin Buffer, Pion Inc.). The acceptor plate and the donor plate were sandwiched together and incubated for 4 h at 37 °C. After incubation, the sandwiched plates were separated, and concentrations were determined using the HPLC method (HPLC Prominence-i LC-2030C, Shimadzu) [36]. Effective permeability (*Pe*) of the compounds was calculated by using the following equation:

(3) $$P_{e}=-\frac{\ln\left(1-\frac{C_{A}}{C_{\mathrm{eq}}}\right)}{S\times \left(\frac{1}{V_{D}}+\frac{1}{V_{A}}\right)\times t}$$

where *Pe* is the effective permeability coefficient (cm/s); V_D_—donor volume; V_A_—acceptor volume; C_eq_—equilibrium concentration, $C_{\mathrm{eq}}=\frac{C_{D}\times V_{D}+C_{A}\times V_{A}}{V_{D}+V_{A}}$,; S—membrane area; t—incubation time (in seconds).

Compounds with *Pe* (10^−6^ cm/s) > 1.5 are classified as high permeation predicted, with *Pe* (*10^−6^ cm/s) < 1.5 classified as low permeation predicted [36,37]. Samples were analyzed in triplicate and the average was reported.

### 2.13. Statistical Analysis

Statistical analysis was performed using GraphPad Prism™ 6.00 software (Graph Pad Software Inc., San Diego, CA, USA). Results were expressed as means ± SEM (standard error of the mean). The median effect concentrations (IC_50_ or IC_0.5_ values) were determined using a concentration–response curve. Statistical differences were calculated using Tukey’s HSD test (STATISTICA 13.0, StatSoft, Kraków, Poland) with significant differences considered at *p* < 0.05.

## 3. Results

The experimental studies carried out as part of this study were divided into three areas: (i) screening of the pharmacological activity of the extract and physodic acid, (ii) evaluation of the composition of the extract, and identification of compounds responsible for its biological activity, and (iii) assessment of the ability to penetrate the blood–brain barrier of physodic acid in the PAMPA-BBB assay.

### 3.1. Screening of Biological Activity

The screening of biological effects of *H. physodes* extract and physodic acid was carried out in relation to (i) anticancer and (ii) neuroprotective properties.

#### 3.1.1. Cytotoxic Activity Against Glioblastoma Cells

**Figure 2:**
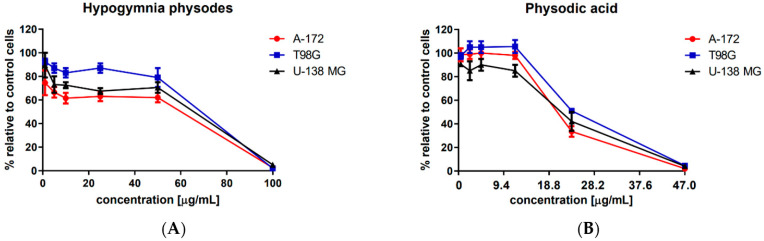
The effect of *Hypogymnia physodes* extract (**A**) and physodic acid (**B**) on the viability of A-172, T98G and U-138 MG cell lines. The mean values ± SEM from three independent experiments with four measurements per assay are presented.

In order to determine the cytotoxicity of physodic acid and *H. physodes* extract, the MTT assay was used. As presented in Figure 2, A-172, T98G and U-138 MG cell lines treated with 0.47–47 μg/mL (1–100 μM) of physodic acid for 48 h responded to the treatment in a very similar manner. Even though the differences were not pronounced, U-138 MG cells were slightly more sensitive to the treatment with physodic acid, especially if it comes to the lowest tested concentration range 0.47–11.75 μg/mL (1–25 μM). The concentration of 0.47–11.75 μg/mL (1–25 μM) did not significantly influence cells viability, however, 23.5 μg/mL (50 μM) concentration reduced the percentage of living cells of A-172, T98G and U-138 MG cell lines to 33%, 51%, and 42%, respectively. The highest tested concentration, namely 100 μM was cytotoxic to all analyzed cell lines. Similarly, *H. physodes* extract at the highest tested concentration of 100 μg/mL led to total cell death of all analyzed cell lines. In contrast, 50 μg/mL and its lower concentrations allowed the survival of ~70%, or even more cells. Interestingly, A-172 cell line was slightly more sensitive to *H. physodes* extract as compared to the other two cell lines. The IC_50_ values are presented in Table 1.

#### 3.1.2. Anti-Hyaluronidase Activity

**Figure 3:**
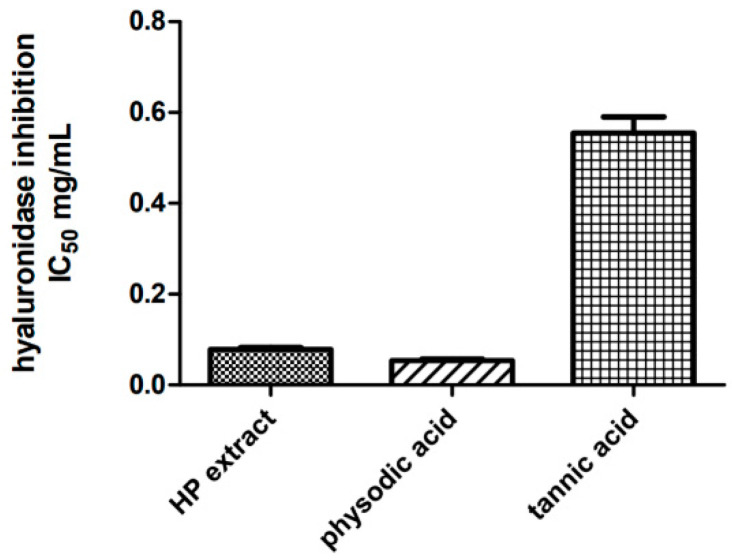
Inhibition of hyaluronidase by *Hypogymnia physodic* extract (HP extract), physodic acid and the references substance tannic acid. Results are presented as IC_50_ values (mg/mL) ± SEM, calculated from five measurments (*n* = 5) obtained from three independent experiments.

To evaluate the inhibitory effect of *H. physodes* extract and physodic acid on hyaluronidase activity, the in vitro method was used. The analysis has shown that both the extract and physodic acid have a high ability to inhibit the enzyme (Figure 3). Physodic acid (IC_50_ = 0.053 mg/mL) was more active than *H. physodes* extract (IC_50_ = 0.078 mg/mL). The compounds were from 6 to 10 times more active than the strong hyaluronidase inhibitor-tannic acid (IC_50_ = 0.554 mg/mL). The results are obtained for the first time for the physodic acid and *H. physodes* extract.

#### 3.1.3. Inhibition of COX-2

The anti-inflammatory activity of *H. physodes* extract and physodic acid via the COX-2 inhibition analysis was estimated. The obtained results showed that among the two tested substances, the extract from *H. physodes* had the high activity of inhibiting the enzyme. *H. physodes* extract inhibited the COX-2 activity at the concentrations of 304 and 456 µg/mL, respectively; however, the concentration of 152 µg/mL showed no inhibitory activity (Table 2). Physodic acid showed no inhibitory activity at the concentrations applied (100, 200 or 300 µg/mL). The results of the study suggest that, in the model of anti-inflammatory activity selected for the experiment, the extract from *H. physodes* had promising properties. At the same time, the solutions of pure physodic acid turned out to be inactive.

#### 3.1.4. Anti-Tyrosinase Activity

**Figure 4:**
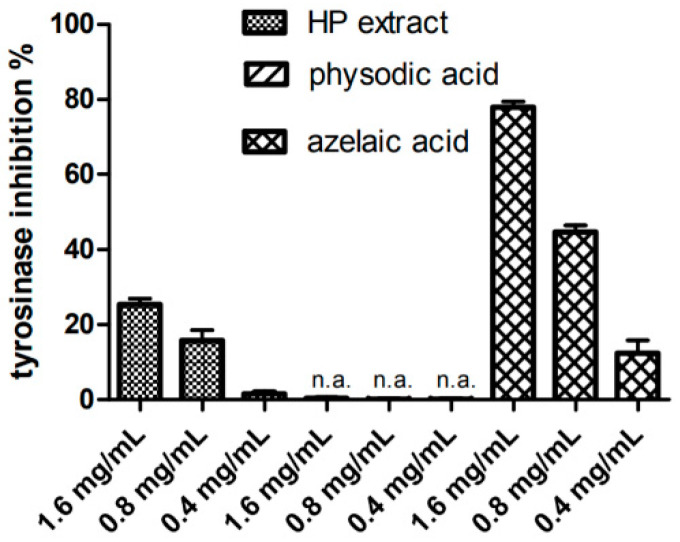
Inhibition of tyrosinase by *Hypogymnia physodes* extract (HP extract), physodic acid and the reference substance azelaic acid. The mean values ± SEM from two independent experiments with two measurements per assay are presented (*n* = 4). “n.a.”—not active.

The inhibition of tyrosinase, which is a popular target in drug research for PD, was estimated using in vitro method with L-DOPA as its substrate. The obtained results showed that the extract inhibits the enzyme in a dose-dependent manner (Figure 4). At a higher concentration (final concentration 1.6 mg/mL), the tested sample inhibited the enzyme by 25%. This activity was only about three times lower as compared to azelaic acid, a potent enzyme inhibitor used as a standard, and tested in the same concentration. The tyrosinase inhibitory activity of *H. physodes* extract, and physodic acid has not yet been described in the literature previously.

#### 3.1.5. Anticholinesterase Activity

To investigate the anticholinesterase effect (anti-AChE and anti-BChE) of *H. physodes* extract and physodic acid Ellman’s analysis was effectuated. Both tested substances indicate a low capacity of inhibition of enzymes (AChE and BChE) (Table 3). Interestingly, the affinity of the tested substances for the type of cholinesterase differed depending on the type of enzyme. Physodic acid inhibited only BChE, while *H. physodes* extract acted solely on AChE.

#### 3.1.6. Antioxidant Activity

**Figure 5:**
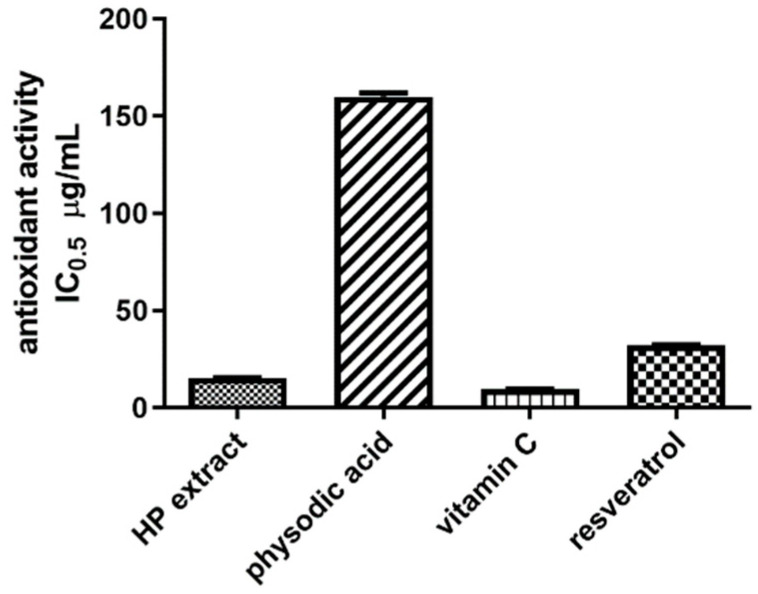
Antioxidant activity of *H. physodes* extract (HP extract), physodic acid, and the reference substances: resveratrol and vitamin C, measured in CUPRAC experiment. Results are presented as IC_0.5_ values (µg/mL) as the mean value ± SEM, the average from *n* = 6 measurements was calculated.

The assessment of antioxidant activity of *H. physodes* extract and physodic acid was performed by using CUPRAC method (not previously used for this purpose). In our study, the IC_0_._5_ value for extract was 15.49 μg/mL and was only 1.5 times lower than the value characterizing vitamin C activity (IC_0.5_ = 9.70 μg/mL). The activity of resveratrol whose multidirectional biological activity results from widely recognized antioxidant properties, was described by the value of IC_0.5_ = 32.07 μg/mL and was almost two times lower than the activity of *H. physodes* extract. The tested IC_0.5_ for physodic acid was 160 µg/mL. Thus, physodic acid had about five times lower antioxidant activity, as compared to resveratrol. The obtained results are presented below in Figure 5.

### 3.2. Phytochemical Analysis

#### 3.2.1. Total Polyphenols Content (TPC)

TPC was also determined in the tested extract using the Folin–Ciocalteu method. *H. physodes* extract was characterized by a significant content of phenolic compounds (289.90 ± 3.68 mg gallic acid equivalent/g of extract), which is equivalent to almost 29% of the compound with the phenolic structure.

#### 3.2.2. High-Performance Liquid Chromatography (HPLC) Analysis

**Figure 6:**
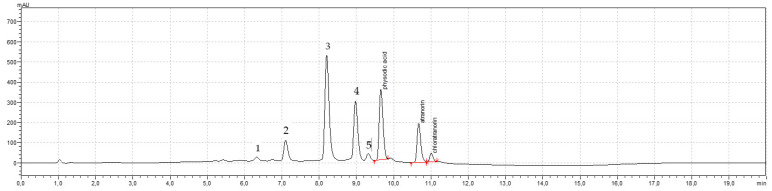
The chromatogram of extract from *Hypogymnia physodes*. Physodic acid, atranorin and chloroatranorin are identified in the extract. Compounds 1, 2, 3, 4, and 5, are characterized by t_R_ = 6.30, 7.10, 8.20, 8.97, and 9.32 min, respectively.

The best separation of the *H. physodes* extract was obtained for atranorin (t_R_ = 10.72 min), chloroatranorin (t_R_ = 11.06 min), and physodic acid (t_R_ = 9.66 min) (Figure 6) which were present in *H. physodes* extract when gradient elution (acetonitrile and 0.5% formic acid) on 5 μm core-shell particles was applied. As a result of the optimization steps, the components of *H. physodes* extract were separated below 12 min.

Peaks were symmetrical, clearly separated from each other (resolution over 1.5). The method was linear from 0.009091 to 0.3 mg/mL (*n* = 5, *R* = 0.9982). The RSD values for intraday and interday precision were below 2%, so the developed method was precise. The method was also accurate (the recovery value was around 100%).

The HPLC analysis of *H. physodes* extract confirmed that it contained 21.09% of physodic acid and 3.58% of atranorin. Chloroatranorin was qualitatively defined in the extract. For this purpose, atranorin as a reference and the mixture of both atranorin (t_R_ = 10.72 min) and chloroatranorin (t_R_ = 11.06 min) were used. Besides, the two peaks with the retention times t_R_ = 8.20 min and t_R_ = 8.97 min strongly suggested the presence in the extract two other main depsidones of *H. physodes*: physodalic acid and 3-hydroxyphysodic acid. This finding is supported by various literature data [38,39,40,41]. In addition, more compounds were detected in the extract. Compounds marked as **1**, **2**, **3**, **4**, and **5**, and characterized by t_R_ = 6.30, 7.10, 8.20, 8.97 m and 9.32 min, respectively. According to the literature data, these are most likely salazinic acid, protocetraric acid or alpha-alectoronic acid, and minimal amount other derivatives of physodic acid (4-O-methylphysodic acid or 2′-O-methylphysodic acid) [40].

### 3.3. Permeability through the Blood–Brain-Barrier (PAMPA-BBB)

**Figure 7:**
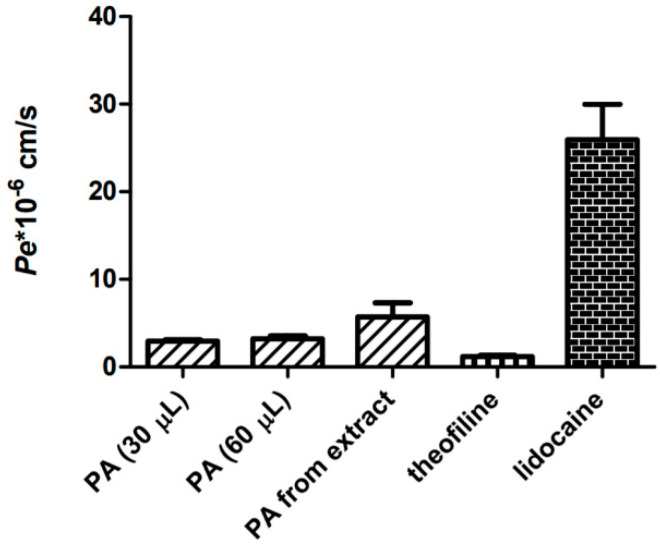
Permeability of pure physodic acid (PA) at different concentrations, physodic acid from extract (PA from extract), and the reference substances: theofiline and lidocaine, examined using PAMPA-BBB model. Results are presented as *Pe* * 10^−6^ cm/s. The mean values ± SEM from three independent experiments are presented (*n* = 3).

**Figure 8:**
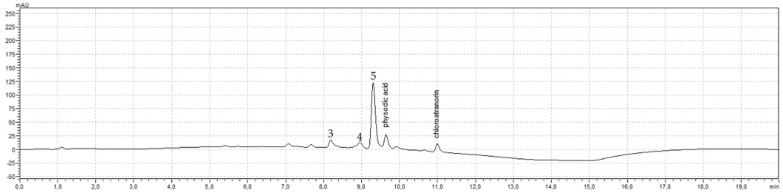
The chromatogram of acceptor solution of extract from *Hypogymnia physodes*. Physodic acid (t_R_ = 9.64 min) and chloroatranorin (t_R_ = 10.99 min) are the identified compounds visible in the acceptor chromatogram. Compounds 3, 4, and 5 are characterized by t_R_ = 8.19, 8.96, 9.31 min, respectively.

For the investigation of the transcellular passive diffusion of physodic acid across the blood–brain barrier, the Parallel Artificial Membrane Permeability Assay PAMPA-BBB method was used. The solution of pure physodic acid, and the *H. physodes* extract were evaluated to determine the effective permeability coefficient (*Pe*). As shown in Figure 7, the permeability of physodic acid from the *H. physodes* extract was higher as compared to physodic acid from its pure compound solution. Moreover, the concentration in donor solution of physodic acid was higher for the pure compound than for the acetone extract (0.45–0.90 and 0.19 mg/mL, respectively). Nevertheless, the permeability coefficient of both tested samples was regarded as high (>1.5 × 10^−6^ cm/s) [36,37]. Thus, both physodic acid itself, as well as physodic acid included in the *H. physodes* extract, can easily pass through the blood–brain barrier via passive diffusion. Furthermore, the chromatogram of the acceptor sample suggests the other compounds can penetrate CNS (Figure 8).

## 4. Discussion

Lichens are a great source of bioactive, phenolic compounds, including depsides, depsidones, anthraquinones, xanthones, and dibenzofuranes. Even though lichens have been used in traditional medicine for ages, the molecular mechanisms responsible for their cellular actions have only recently been evaluated and understood. In this regard, it has been shown that lichen-derived secondary metabolites can act as e.g., anticancer, antimicrobial, antiviral, anti-inflammatory, or antioxidant agents [17,18,19,20,42]. *H. physodes* is an especially interesting lichen, since it is widely distributed worldwide, providing a powerful natural source of compounds with potential pharmaceutical applications. In our previous study, we showed that *H. physodes* derived depsidone-physodic acid, was cytotoxic to HCT116 and DLD-1 colorectal cancer cells [26]. Moreover, it inhibited the expression of β-catenin-dependent genes and attenuated cell migration [26]. We also showed that physodic acid and *H. physodes* extract was cytotoxic to MDA-MB-231, MCF-7 and T-47D breast cancer cell lines and that *H. physodes* extract possesses significant antioxidant activity [43].

Considering the attractive biological potential of *H. physodes*, we hypothesized that *H. physodes* acetone extract and its major components, physodic acid might possess biological properties important for glioblastoma treatment or neurodegenerative diseases treatment. The few studies in this area prompted us to undertake the work that attempts to fill this gap.

Glioblastoma is the most common primary brain tumor in adults [44]. Owing to the BBB, which restricts the infiltration of most antitumor drugs into the CNS, the treatment of this deadly tumor involves maximal surgical resection, radiotherapy, and chemotherapy with temozolomide [44]. However, despite the best possible treatment, tumor recurrence is inevitable, so novel drug candidates or compounds that could ameliorate the treatment are urgently needed. Thus, we wanted to determine if physodic acid and *H. physodes* extract are cytotoxic to glioblastoma cell lines. Even though no clinical study has been conducted yet, there are a number of in vitro and in vivo studies demonstrating anticancer effects of lichen metabolites, including physodic acid [20]. In this study, three glioblastoma cell lines were used, namely A-172, T98G, and U-138 MG. The first mentioned cell line is regarded as temozolomide sensitive, whereas, the last two are temozolomide resistant [45]. Our results confirmed that physodic acid and *H. physodes* extract dose-dependently reduced the viability of all three glioblastoma cell lines, regardless of their temozolomide sensitivity. Physodic acid significantly reduced the viability of the tested cells at 23.5 µg/mL (50 µM) concentration, whereas *H. physodes* extract led to total cell death at 100 µg/mL. However, in regard to *H. physodes* extract, we observed a decrease in cell viability even with the lowest concentrations tested (1–10 μg/mL), in particular in A-172 cell line. This phenomenon can potentially be explained by the fact that *H. physodes* extract, as shown in our HPLC analysis, contains not only physodic acid, but also various other chemicals, including atranorin, chloroatranorin, physodalic acid, and 3-hydroxyphysodic acid, and most likely also salazinic acid, protocetraric acid or alpha-alectoronic acid. The minimal amount of other derivatives of physodic acid (4-O-methylphysodic acid or 2′-O-methylphysodic acid) can also be present, influencing glioblastoma cell viability. In a study by Emsen et al. [22], cell viabilities of rat neuron cell line PRCC and human glioblastoma U87MG cells exposed to different concentrations of physodic acid were determined. The obtained IC_50_ values were 698.19 and 410.72 mg/L for PRCC cell line, and U87G cell lines, respectively, showing higher sensitivity of glioblastoma cells as compared to noncancerous neurons. Importantly, no cytotoxic effects were observed for physodic acid in a study on noncancerous astrocytes [46]. Furthermore, in another study, conducted on murine neuroblastoma Neuro2A cells, lichen-derived secondary metabolites including physodic acid and atranorin, displayed an important increase in neurite outgrowth [47]. Nevertheless, the potential impact of *H. physodes* extract on noncancerous astrocytes and neurons requires further investigation.

Inhibition of certain enzymes is regarded as one of the therapeutic strategies for the management of cancer. Hyaluronidase-hyaluronic acid degrading enzyme is highly expressed in patients with malignant glioma [48]. It produces low molecular weight hyaluronic acid fragments, associated with enhanced invasion and increased tumor growth [9,10]. Thus, in this study we wanted to verify if physodic acid and *H. physodes* extract are able to inhibit hyaluronidase. Our results show that both analyzed substances can strongly inhibit hyaluronidase enzymatic activity, however, physodic acid was more active than *H. physodes* extract. Importantly, both of them were from 6 to 10 times more active than the potent inhibitor of hyaluronidase-tannic acid. Research on the ability to inhibit hyaluronidase by lichen secondary metabolites is minimal. The available results describe the activity only of usnic acid, the most commonly found secondary metabolite in lichens, which was lower than the tannic acid used as the standard [49]. No other results regarding lichen secondary metabolites or lichen extracts have been reported in the literature so far.

Our next goal was to establish the impact of physodic acid and *H. physodes* extract on COX-2 enzymatic activity. COX-2 is another overexpressed enzyme with a key function in glioblastoma. The molecular mechanisms by which COX-2 promotes tumorigenesis are not fully clear, but it is suggested that prostaglandin E2 (PGE2), produced by COX-2, facilitates tumor activities, including tumor cell adhesion, proliferation, migration, angiogenesis, immunosuppression, and metastasis [11]. Our study shows that *H. physodes* extract strongly inhibits COX-2 enzymatic activity, however, it is not physodic acid that exerts this action. Additionally, atranorin, one of the substances present in the extract, was not active at the chosen concentration of 1 mg/mL (unpublished data). Thus, we can speculate that the COX-2 inhibitory activity of *H. physodes* extract results either from the presence of physodic acid derivatives or potentially also synergistic effects of its compounds. This issue requires further investigation.

Even though we did not observe physodic acid COX-2 inhibitory activity, it is important to note that using pharmacophore models Bauer et al. [50] showed that this lichen metabolite inhibits microsomal prostaglandin E2 synthase-1. It is an inducible enzyme that is upregulated at various pathophysiological stages, including glioblastoma, and in conjunction with the inducible COX-2, produces massive amounts of PGE2 [51].

Apart from cancer, the involvement of COX-2 in neurodegenerative processes has also been established. Neuroinflammatory processes involving COX-2 overexpression and elevated PGE2 levels have been associated with several neurodegenerative diseases, including AD, PD, and amyotrophic lateral sclerosis (ALS) [52]. Moreover, another enzyme-tyrosinase, oxidizing excess dopamine to produce dopamine quinones, highly reactive species which induce neural damage and cell death, was also linked to PD and other neurodegenerative diseases. Our study provides evidence that *H. physodes* extract strongly inhibits not only COX-2, but also tyrosinase. Similar results were obtained by Higuchi et al. [53], who found that *H. physodes* methanol extract from in vitro culture tissue strongly inhibited tyrosinase activity. Moreover, the mycobiont of *H. physodes* showed higher inhibitory action (55.3%) as compared to photobiont (22.4%). In our study, we did not find any inhibitory activity of physodic acid on tyrosinase activity; therefore, we can speculate that other secondary metabolites present in this lichen are responsible for this action. However, no studies related to this issue were, to our best knowledge, published so far.

In our study, we also found a small influence of physodic acid and *H. physodes* extract on AChE and BChE activity. Both enzymes are druggable targets in AD. Thus, their inhibiton by the lichen compounds would be beneficial. Such effects were observed in a study of Stojanović et al. [54], who analyzed extract of *Hypogymnia tubulosa*. In this study, the extract inhibited cholinesterase to the extent of 23.6% at a concentration of 10 mg. In comparison, at a concentration of 1 mg it showed a weak activation effect on cholinesterase to the extent of 3.3%. On the other hand, Reddy et al. [46], similarly to our study, did not find any AChE activity of physodic acid. Further research is needed to fully elucidate the impact of physodic acid on AChE and BChE activity.

The level of ROS in our body is kept at a constant level. However, excess free radicals cross the cell membrane, attacking sensitive glial cells and neurons, leading to irreversible damage important in degenerative changes of brain [7,8]. In our study, the *H. physodes* extract (a mixture of depsides and depsidones) had high antioxidant activity, higher (approx. 10 times) than the pure physodic acid. Studies on the antioxidant activity of *H. physodes* extracts, with the use of other than the CUPRAC method [19,43,55] confirm the high activity of the examined extract. Taking into consideration that both physodic acid and *H. physodes* extract show high antioxidant activities, further research, aiming at the determination of the chemical composition of the extract, was undertaken.

The HPLC analysis of acetone extract of *H. physodes* confirmed that it contained physodic acid and atranorin and as well as the other compounds, probably chloroatranorin, physodalic acid, and 3-hydroxyphysodic acid. The percentage of physodic acid and atranorin in the acetone extracts of *H. physodes* from Jastrzębsko Stare, Poland were 21.09% and 3.58%, respectively. On the other hand, Solhaug reported the concentration of around 38% of physodic acid and 2.75% of atranorin in the acetone extract from *H. physodes* collected from Jeløya, Norway. The differences in the concentration values of detected substances are closely connected with the geographic region from which the samples came from [56].

A review of the literature shows that some natural substances can pass into the CNS by passive transport. They include, among others, quercetin and resveratrol, as well as lovastatin, which under natural conditions is produced by the fungus *Pleurotus ostreatus* [57,58]. There are no studies on the permeability of physodic acid through the blood–brain barrier using the PAMPA-BBB model. Our tests confirmed that both physodic acid as a single substance as well as physodic acid from *H. physodes* extract are both able to diffuse through the BBB. The permeability coefficient of both analyzed substances was regarded as high [36,37], meaning they can easily reach the CNS and potentially also brain tumor mass. Thus, they could successfully be used in the treatment of glioblastoma or neurodegenerative diseases.

## 5. Conclusions

The lack of effective therapy of neoplastic brain diseases and degenerative changes in the CNS indicates the need to search for new substances with a therapeutic potential. Our attempt to evaluate the antiglioma properties demonstrated cytotoxicity of *H. physodes* extract and physodic acid against glioblastoma cells. The ability to inhibit both hyaluronidase and COX-2 activity by *H. physodes* extract and its active compound suggest properties that may lower tumor formation rate. The ability to inhibit tyrosinase and cholinesterase activity, and high antioxidant properties confirm the neuroprotective properties of the extract. Our study confirmed the anticancer, chemopreventive and neuroprotective properties of physodic acid and *H. physodes* extract. Nevertheless, in order to fully confirm their usefulness in the prevention and treatment of brain tumors and neurodegenerative diseases, more studies are needed, especially using cell cultures and animal models.

## Figures and Tables

**Table 1:** The IC_50_ of cells viability after *Hypogymnia physodes* extract or physodic acid exposition.

Type of Cells	*H. Physodes* Extract	Physodic Acid
	IC_50_ [µg/mL]	IC_50_ [µg/mL (µM)]
A-172	61.37 ± 5.19	19.95 ± 0.59 (42.41 ± 1.25)
T98G	72.15 ± 4.33	23.79 ± 0.51 (50.57 ± 1.09)
U-138MG	68.36 ± 1.58	21.51 ± 1.98 (45.72 ± 4.20)

IC_50_ is the mean values ± SEM calculated from the curve plotted using three independent experiments with four measurements per assay.

**Table 2:** Inhibition of cyclooxygenase-2 (COX-2) by *Hypogymnia physodes* extract (*H. physodes* extract) and physodic acid.

Concentration (Final Concentration)		Inhibition of COX-2	
*H. physodes* extract	Physodic acid	*H. physodes* extract	Physodic acid
152 µg/mL	100 µg/mL	n.a.	n.a.
304 µg/mL	200 µg/mL	28.60% ± 2.4%	n.a.
456 µg/mL	300 µg/mL	52.40% ± 1.1%	n.a.

The mean values ± SEM from four independent measurements, “n.a.”—not active.

**Table 3:** Inhibition of acetylcholinesterase (AChE) and butyrylcholinesterase (BChE) by *Hypogymnia physodes* extract (*H. physodes* extract) and physodic acid comparing with the reference substances.

Materials for Studies	Inhibition of Enzyme	
	AChE	BChE
***H. physodes* extract** 72.5 µg/mL	9.6 ± 0.1%	n.a.
**Physodic acid** 72.5 µg/mL (154 µM)	n.a.	8.1 ± 0.2%
Inhibition similar to:	**eserine** at 0.12 ± 0.007 μg/mL	**eserine** at 0.019 ± 0.001 μg/mL
	**donepezil** at 0.30 ± 0.013 μg/mL	**donepezil** at 0.026 ± 0.014 μg/mL

The mean values ± SEM from eight independent measurements “n.a.”—not active.

## Data Availability

The data supporting reported results can be found in Department of Pharmacognosy, Poznan University of Medical Sciences; Department of Pharmaceutical Biochemistry, Poznan University of Medical Sciences; Department of Biotechnology, Microbiology and Human Nutrition, University of Life Sciences in Lublin.
